# Effect of Different Glucose Levels and Glycation on Meningioma Cell Migration and Invasion

**DOI:** 10.3390/ijms251810075

**Published:** 2024-09-19

**Authors:** Philipp Selke, Christian Strauss, Rüdiger Horstkorte, Maximilian Scheer

**Affiliations:** 1 Institute for Physiological Chemistry, Medical Faculty, Martin-Luther-University Halle-Wittenberg, 06114 Halle (Saale), Germany; philipp.selke@uk-halle.de (P.S.);; 2 Department of Neurosurgery, Medical Faculty, Martin-Luther-University Halle-Wittenberg, Ernst-Grube-Str. 40, 06120 Halle (Saale), Germany

**Keywords:** meningioma, glycation, glucose, migration, invasion, sialic acid, focal adhesion, integrin β1, merlin, MGO

## Abstract

Meningiomas are predominantly benign tumors, but there are also malignant forms that are associated with a poor prognosis. Like almost all tumors, meningiomas metabolize glucose as part of aerobic glycolysis (Warburg effect) for energy supply, so there are attempts to influence the prognosis of tumor diseases using a glucose-reduced diet. This altered metabolism leads to so called hallmarks of cancer, such as glycation and glycosylation. In this study, we investigated the influence of low (3 mM), normal (5.5 mM) and high glucose (15 mM) on a malignant meningioma cell line (IOMM-Lee, WHO grade 3). In addition, the influence of methylglyoxal, a by-product of glycolysis and a precursor for glycation, was investigated. Impedance-based methods (ECIS and RTCA) were used to study migration and invasion, and immunoblotting was used to analyze the expression of proteins relevant to these processes, such as focal adhesion kinase (FAK), merlin or integrin ß1. We were able to show that low glucose reduced the invasive potential of the cells, which was associated with a reduced amount of sialic acid. Under high glucose, barrier function was impaired and adhesion decreased, which correlated with a decreased expression of FAK.

## 1. Introduction

Meningioma is the most common intracranial tumor, and its incidence typically increases with age [1,2,3]. The majority of meningiomas are benign (World Health Organization (WHO) grade 1). However, there are atypical (WHO grade 2) and malignant (WHO grade 3) types of meningiomas, which are often prone to recurrence [4]. Due to the invasive nature of malignant meningiomas in particular, therapeutic options are limited and the prognosis is unfavorable [5].

Like almost all tumors, meningiomas consume large amounts of glucose as their primary energy source, as they metabolize glucose primarily to lactate during anaerobic glycolysis. This is known as the Warburg effect [6]. This altered (anaerobic) energy metabolism is one of the “hallmarks of cancer” [7,8]. High glucose levels therefore provide a favorable environment for tumor cells. Typically, glucose tolerance declines with age, resulting in higher serum glucose levels [9,10].

Consistent with this, some studies have shown a positive association between elevated serum glucose levels and the risk of developing meningioma [11,12,13,14]. However, there are also conflicting statements regarding the relationship between diabetes mellitus, which is associated with elevated glucose levels and the risk of developing meningioma, suggesting a positive [15,16] or negative [17] association.

When patients with meningioma also have type 2 diabetes mellitus (T2DM), this population has been shown to have decreased overall survival after surgical resection of WHO grade 1 meningioma [18]. A hyperglycemic environment also results in increased cell proliferation and extracellular matrix (ECM) stiffness, and is inhibited when lowering the blood glucose level in breast cancer [19]. Increased ECM stiffness and high glucose promotes epithelial–mesenchymal transition (EMT) in breast tumor cells [20,21]. EMT is associated with a loss of cell-to-cell contacts and increased cell migration and invasion [22]. In T2DM, an increase in the permeability of the blood vessels may occur through an increase in advanced glycation end products and vascular inflammation [23], promoting tumor cell adhesion, transendothelial tumor cell movement and the development of metastases [24].

In addition, in a glucose-based PET (fluorodeoxyglucose positron emission tomography; FDG-PET) study in patients with meningioma, the rate of glucose metabolism was shown to correlate with tumor growth and recurrence [25]. Because this phenomenon is known to occur in other tumors, such as high-grade gliomas, attempts are being made to improve prognosis by lowering glucose levels as part of a ketogenic diet [26,27].

Another phenomenon observed due to altered and increased glycolysis in tumors is the accumulation of glycolysis by-products. One such by-product is methylglyoxal (MGO). This is approximately 20,000 times more reactive than glucose and has been discussed as a possible link between diabetes, elevated serum glucose levels and cancer [28], as diabetic and elderly individuals have elevated MGO concentrations [29]. In addition, MGO is considered a tumor-promoting agent as it can enhance the growth and invasiveness of tumor cells [30,31]. Nearly 0.1–0.4% of glucose is converted to MGO during glycolysis as a regular by-product of dihydroxyacetone phosphate or glyceraldehyde-3-phosphate [32]. MGO reacts primarily with proteins (via arginine, lysine and cysteine residues) or, to a lesser extent, with DNA or lipids to form advanced glycation end products (AGEs) [33,34,35]. This non-enzymatic reaction between the carbonyl groups of dicarbonyls (such as MGO or glyoxal) or sugars (such as glucose or fructose) and the amino groups of proteins is called glycation [36,37]. Electrophilic carbonyl groups of glucose or other reactive sugars react with free amino groups of amino acids to form an unstable Schiff base. This reaction is known as the classical Maillard reaction. Further rearrangement leads to the formation of a more stable ketoamine (Amadori product). The formation of Schiff bases and Amadori products are reversible reactions. In later reactions, they form irreversible adducts or protein cross-links [36,38]. The process of AGE formation affects all proteins, including cell adhesion molecules or receptors and extracellular matrix proteins [39,40].

In addition to glycation, many tumors also exhibit altered glycosylation. Both are post-translational modifications [41,42], with glycosylation being an enzymatic process [43]. Sialylation, as a form of glycosylation, describes the attachment of sialic acids (Sia) to lipids (e.g., gangliosides) or proteins (e.g., neural cell adhesion molecule (NCAM)) via sialyltransferases (ST) [44]. The reaction products, called glycans, are involved in fundamental molecular and cell biological processes that occur in cancer, such as cell signaling and communication, tumor cell dissociation and invasion, cell–matrix interactions, tumor angiogenesis, immunomodulation and metastasis formation. In recent years, there has been a growing interest in these processes in brain tumors [45,46,47].

In previous studies by our group, using benign and malignant meningioma cell lines (WHO grade 1 and 3), we showed that physiological concentrations of MGO had no effect on metabolic activity. However, we observed increased AGE formation in both lines. In addition, there were different effects on the invasiveness of benign and malignant cells. The altered expression of cadherin correlated with an altered expression of sialyltransferases [48,49]. These belong to the glycosyltransferases, which are responsible for tumor glycosylation, among others [50].

These works were performed under standard cell culture conditions with respect to glucose concentration (25 mM), which corresponds to a highly derailed blood glucose level in humans [51].

In the present study, we focused on the malignant cell line (IOMM-Lee, WHO grade 3) and the influence of low (3 mM), normal (5.5 mM) and high (15 mM) glucose concentrations with and without additional glycation by MGO (0.3 mM). We used impedance-based methods to measure migration and invasion. In addition, enzymes such as FAK (focal adhesion kinase) or integrins were quantified using Western blot.

Low glucose levels were associated with a reduction in cell viability and led to a significant decrease in invasive potential and sialic acid levels. High glucose levels with additional glycation also reduced invasive behavior, but did not affect sialic acid levels. At high glucose levels, barrier function was disrupted, adhesion was reduced and cell confluence was increased. The expression of FAK was significantly reduced at high glucose levels.

The results of this study suggest that an intentional low-glycemic diet may improve the outcome of patients with malignant meningioma by positively influencing tumor growth and potential.

## 2. Results

### 2.1. Low Glucose with and without Additional Glycation Interferes with Cell Viability

MTT assays were used to determine whether low, normal or high glucose concentrations with or without MGO treatment affected cell viability.

**Figure 1:**
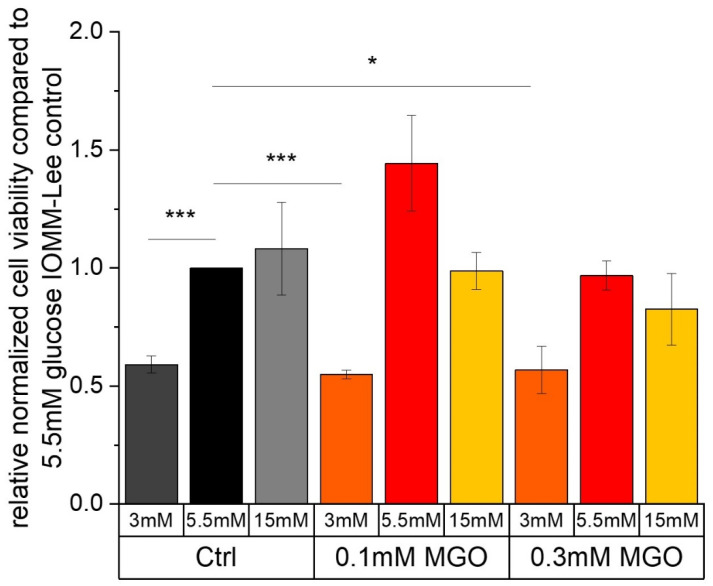
Cell viability of IOMM-Lee cell line in different glucose levels with and without MGO. MTT cell viability assay was performed after 24 h treatment with different glucose concentrations (3 mM, 5.5 mM and 15 mM) with or without MGO (0.1 mM, 0.3 mM). Graph shows relative cell viability of IOMM-Lee cell line treated with different glucose concentrations with or without MGO (0.1 mM, 0.3 mM), normalized to 5.5 mM glucose. Statistical analysis was performed using *t*-test and error bars represent SD (n = 3, *p* < 0.05 = *, *p* < 0.005 = ***).

Figure 1 shows the cell viability of the malignant meningioma cell line IOMM-Lee after treatment with different glucose levels. Low glucose treatment (0.591 ± 0.036; *p* = 0.0039) significantly decreases cell viability compared to normal glucose treatment.

Additional treatment with MGO results in a significant reduction in the cell viability of the IOMM-Lee cell line at low glucose compared to the normal glucose control. Exposure to 0.1 mM MGO (3 mM 0.1 mM MGO: 0.55 ± 0.018; *p* = 0.0008) results in a higher reduction in cell viability compared to the next highest concentration of 0.3 mM MGO (0.569 ± 0.1; *p* = 0.0261). We also observe a slight but not significant increase in cell viability with normal glucose and additional treatment with 0.1 mM MGO (1.444 ± 0.202; *p* = 0.0895).

Other treatments show no significant effect on cell viability after 24 h in the malignant cell line.

### 2.2. Migration Is Differently Affected by Glucose Level with or without MGO

In order to investigate whether glucose, with or without additional glycation, has an effect on migration, we performed an ECIS assay.

**Figure 2:**
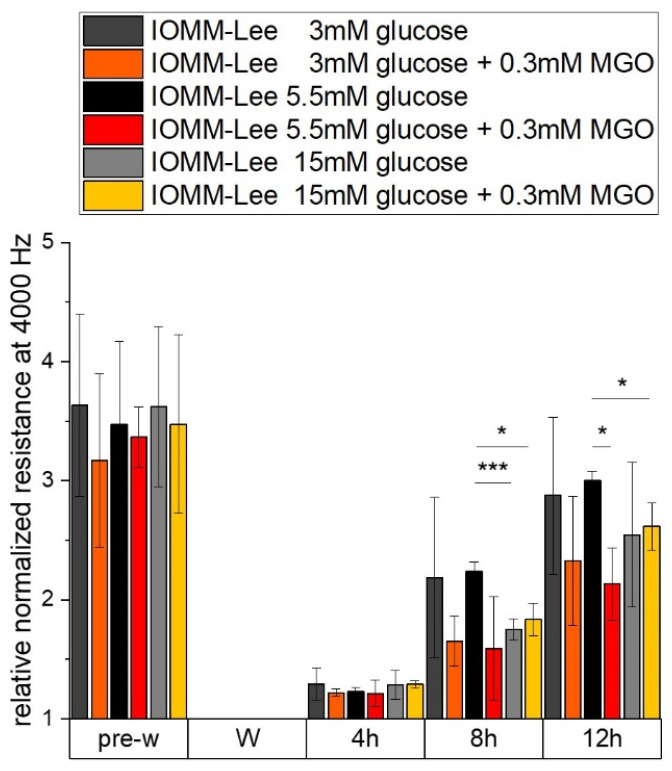
Resistance of IOMM-Lee cells in different glucose levels with and without MGO. Cells were grown in arrays to confluence and treated with different glucose concentrations (3 mM, 5.5 mM and 15 mM) with or without MGO (0.3 mM). Wounding started 12 h after treatment. Immediately before wounding (pre-w), wounding (W) and time points after W (4 h, 8 h, 12 h). Each treatment was normalized to wounding (W). Graph shows relative normalized resistance at 4000 Hz of IOMM-Lee cells. Statistical analysis was performed using *t*-test and error bars represent SD (n = 3, *p* < 0.05 = *, *p* < 0.005 = ***).

**Figure 3:**
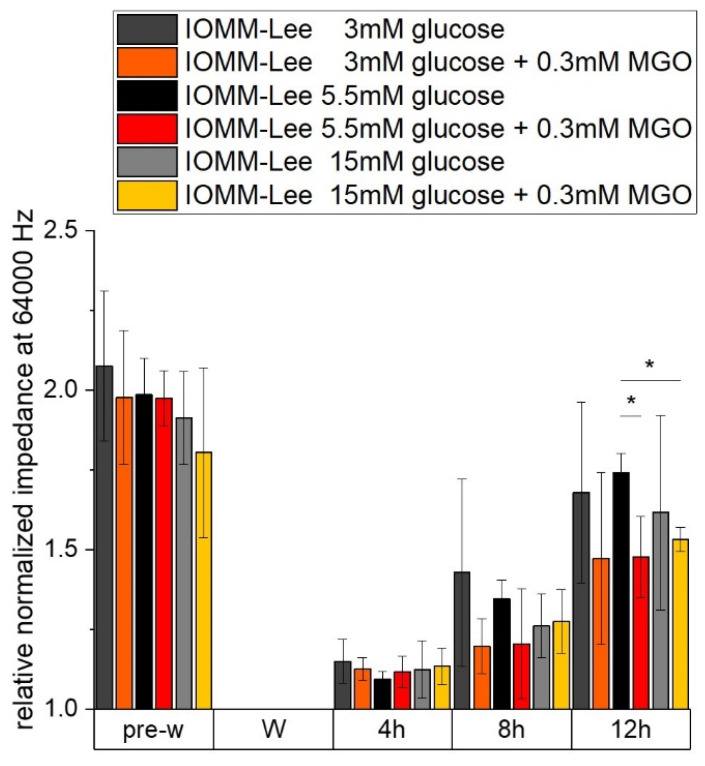
Impedance of IOMM-Lee cells in different glucose levels with and without MGO. Cells were grown in arrays to confluence and treated with different glucose concentrations (3 mM, 5.5 mM and 15 mM) with or without MGO (0.3 mM). Wounding started 12 h after treatment. Immediately before wounding (pre-w), wounding (W) and time points after W (4 h, 8 h, 12 h). Each treatment was normalized to wounding (W). Graph shows relative normalized impedance at 64,000 Hz of IOMM-Lee cells. Statistical analysis was performed using *t*-test and error bars represent SD (n = 3, *p* < 0.05 = *).

The measured resistance of the differently treated IOMM Lee cells is shown in Figure 2. All treatments have nearly the same relative normalized resistance at 4000 Hz before wounding. Resistance can provide evidence of cell–cell contacts that are negatively affected by 15 mM glucose with (1.835 ± 0.136; *p* = 0.016) and without (1.751 ± 0.09; *p* = 0.0046) additional MGO treatment 8 h after wounding. At 12 h after wounding, only additional treatment with MGO has effects in 5.5 mM glucose (2.132 ± 0.305; *p* = 0.024) and 15 mM glucose (2.615 ± 0.196; *p* = 0.047) treatments. These effects were also observed in Figure 3.

The measured impedance of the IOMM-Lee cells, which can give an indication of adhesion, spreading and confluence, is shown in the next graph (Figure 3). The relative normalized impedance at 64,000 Hz before wounding is almost the same in all samples. Except for the high glucose with additional MGO treatment (1.804 ± 0.266; *p* = 0.224), we observed a lower but not significant impedance compared to the impedance of the other pre-wounding measurements of normal glucose (1.985 ± 0.114).

At 12 h after wounding, we observe a lower signal in normal glucose (1.478 ± 0.127; *p* = 0.041) and high glucose (1.532 ± 0.038; *p* = 0.01)—both with additional MGO treatment compared to normal glucose without additional treatment.

**Figure 4:**
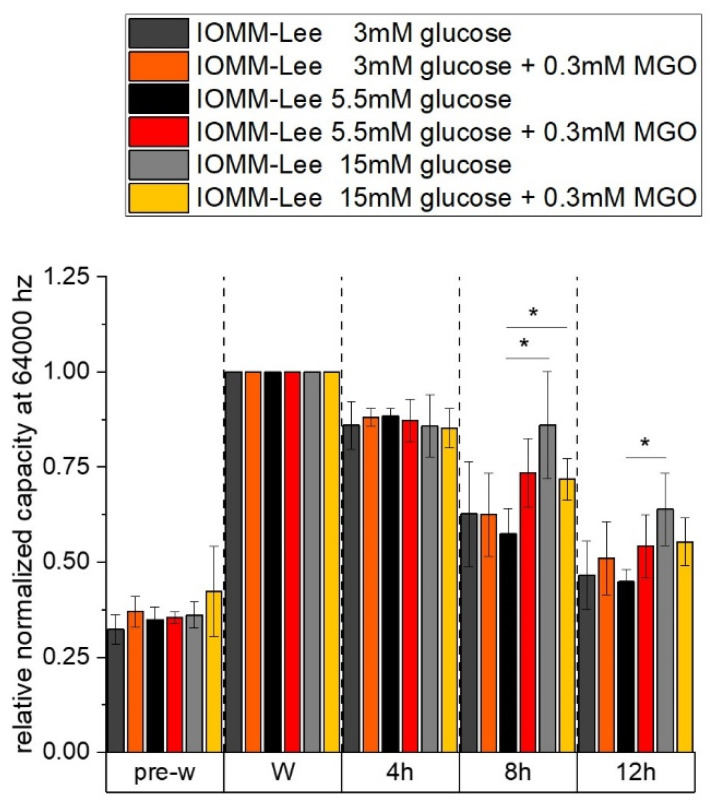
Capacity of IOMM-Lee cells in different glucose levels with and without MGO. Cells were grown in arrays to confluence and treated with different glucose concentrations (3 mM, 5.5 mM and 15 mM) with or without MGO (0.3mM). Wounding started 12 h after treatment. Immediately before wounding (pre-w), wounding (W) and time points after W (4 h, 8 h, 12 h). Each treatment was normalized to wounding (W). The graph shows the relative normalized capacity at 64,000 Hz of IOMM-Lee cells. Statistical analysis was performed using *t*-test and error bars represent SD (n = 3, *p* < 0.05 = *).

The relative normalized capacity at 64,000 Hz of different glucose with and without MGO treatment in IOMM Lee cells is shown in the graph in Figure 4. The capacity indicates the spreading and confluence of the cells. Measurements taken before the wounding process show the same results as we observed in the impedance measurements.

At 8 h after wounding, measurements show a higher capacity in 15 mM with (0.718 ± 0.0541; *p* = 0.0394) and without (0.861 ± 0.141; *p* = 0.0427) MGO compared to the 5.5 mM control (0.575 ± 0.066). The higher capacity is an indication of lower confluence or spreading behavior. In normal glucose with glycation, there is an increase but it is not significant (0.735 ± 0.089; *p* = 0.059).

At 12 h after wounding, we see a higher capacity only in high glucose (0.639 ± 0.095; *p* = 0.047) compared to normal glucose (0.448 ± 0.032). With additional glycation, we observe a slight increase but no significant capacity in high glucose (0.553 ± 0.063; *p* = 0.064) compared to 5.5mM glucose.

### 2.3. Expression of Focal Adhesion and Phosphorylated Focal Adhesion Is Not Affected by Glucose Level but by Glycation

To determine whether the effects of treatment on migration also showed differences in focal adhesion expression, immunoblots were performed.

**Figure 5:**
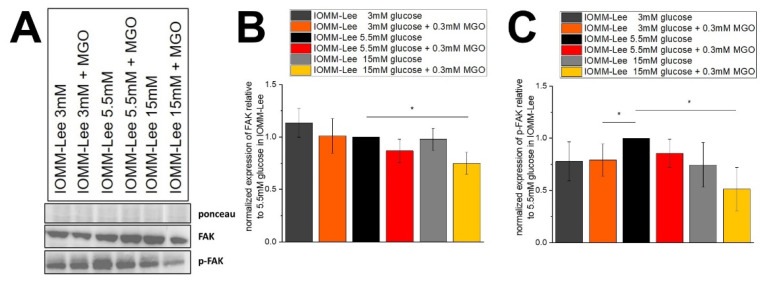
Expression of focal adhesion kinase in IOMM-Lee cells in different glucose levels with and without MGO. Cells were treated with different glucose levels with and without additional treatment with 0.3 mM MGO. (**A**) representative immunoblot of FAK, p-FAK with Ponceau staining as loading control. (**B**) quantification with normalized expression of FAK relative to 5.5 mM glucose in IOMM-Lee. (**C**) quantification with normalized expression of p-FAK relative to 5.5 mM glucose in IOMM-Lee. Statistical analysis was performed using *t*-test and error bars represent SD (n = 4, *p* < 0.05 = *).

Figure 5A shows representative immunoblots of FAK and p-FAK. Figure 5B shows the quantification of FAK expression in treated IOMM-Lee cells. We observed a slightly decreased FAK expression in normal glucose with additional MGO treatment (0.868 ± 0.112; *p* = 0.067) compared to normal glucose control. Low glucose treatment shows a slight upregulation of FAK expression, but it is not significant (1.134 ± 0.136; *p* = 0.149). The addition of MGO to low glucose has no effect on FAK expression. The 15 mM glucose with MGO treatment shows a significantly decreased (0.748 ± 0.105; *p* = 0.04) signal in the expression of FAK in IOMM-Lee cells.

Figure 5C shows that additional treatment with MGO in low (0.793 ± 0.152; *p* = 0.0497) and high glucose (0.513 ± 0.209; *p* = 0.041) resulted in decreased expression of p-FAK in three independent experiments. The expression of p-FAK is even slightly but not significantly decreased in normal glucose with MGO (0.857 ± 0.136; *p* = 0.083) and high glucose without MGO (0.745 ± 0.213; *p* = 0.065) compared to normal glucose control.

### 2.4. Glucose Levels Correlate with Integrin β1 Expression

Immunoblotting was used to determine whether cell surface proteins that influence invasion behaved under the different glucose levels.

**Figure 6:**
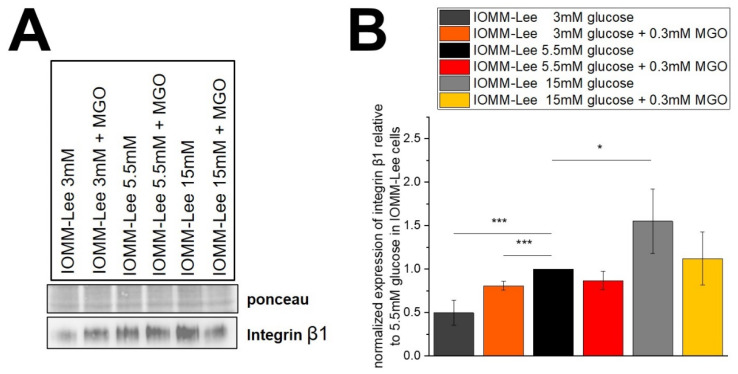
Expression of integrin β1 in IOMM-Lee cells in different glucose levels with and without MGO. Cells were treated with different glucose levels with and without additional 0.3 mM MGO. (**A**) representative immunoblot of integrin β1 with Ponceau staining as loading control. (**B**) quantification with normalized expression of integrin β1 relative to 5.5 mM glucose in IOMM-Lee. Statistical analysis was performed by *t*-test and error bars represent SD (n = 4, *p* < 0.05 = *, *p* < 0.005 = ***).

Figure 6A shows representative immunoblots of integrin β1 expression in IOMM-Lee cells treated with different glucose levels with and without 0.3 mM MGO. Quantification is shown in the graph (Figure 6B). Integrin β1 expression is decreased in low glucose with and without MGO treatment compared to the normal glucose level (3 mM: 0.496 ± 0.145; *p* = 0.0046, 0.3 mM MGO: 0.807 ± 0.050; *p* = 0.0035). The high glucose level of 15 mM shows a significantly increased expression without additional glycation (1.55 ± 0.372; *p* = 0.042). Treatment with MGO in high glucose also shows an increased expression. However, it is not significant (1.121 ± 0.306; *p* = 0.271). Normal glucose with MGO treatment shows a decreased but not significant change in the expression of integrin β1 (0.868 ± 0.104; *p* = 0.057) in IOMM-Lee cells.

### 2.5. Glucose Levels and MGO Treatment Have No Effect on Merlin Expression

To verify whether merlin/NF2 is affected by different glucose levels and/or glycation and thus has an effect on survival, we performed immunoblotting.

**Figure 7:**
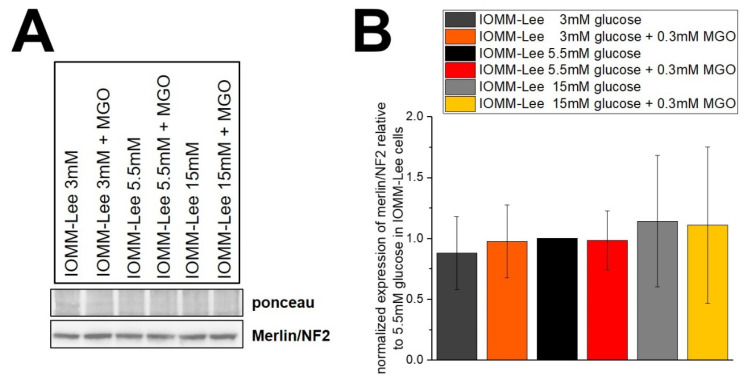
Expression of merlin/NF2 in IOMM-Lee cells in different glucose levels with and without MGO. Cells were treated with different glucose levels with and without additional 0.3 mM MGO. (**A**) representative immunoblot of merlin/NF2 with ponceau staining as loading control. (**B**) quantification with normalized expression of merlin/NF2 relative to 5.5 mM glucose in IOMM-Lee. Statistical analysis was performed using *t*-test and error bars represent SD (n = 5).

The representative blot (Figure 7A) shows merlin/NF2 expression in IOMM-Lee cells treated with different glucose levels with and without 0.3 mM MGO. Figure 7B shows the quantification of five independent experiments. Low and high glucose show a slight down- and upregulation of merlin/NF2 expression, but without being significant. The additional treatment with MGO also shows no significant differences, with a trend towards lower expression in low glucose and higher expression in high glucose.

### 2.6. Low Glucose Is Associated with Reduced Invasive Potential

**Figure 8:**
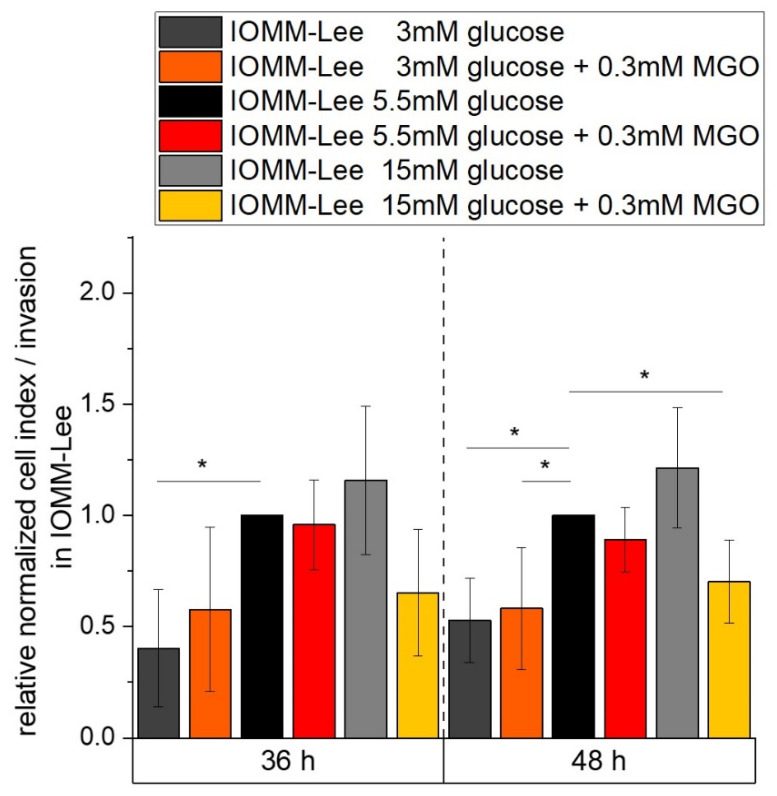
Invasive potential of IOMM-Lee in different glucose levels with and without MGO. Invasive behavior of malignant meningioma cells was measured with RTCA over 48 h. Graph shows cell index/invasion of IOMM-Lee cell line treated with different glucose concentrations with and without 0.3mM MGO over 48 h. Statistical analysis was performed using *t*-test and error bars represent SD (n = 4; *p* < 0.05 = *).

We performed invasion assays with the RTCA to analyze the effect of different glucose levels without or with additional glycation on the invasive potential of meningioma cells (Figure 8).

At 36 h, low glucose results in a strong decrease (0.403 ± 0.264; *p* = 0.043) in invasive potential compared to the normal glucose control. After 48 h of analysis, the low glucose level shows an approximately 2-fold decrease in impedance measurement (0.528 ± 0.189; *p* = 0.017) with an additional 0.3 mM MGO treatment (0.582 ± 0.274; *p* = 0.039) compared to the normal glucose control of IOMM-Lee cells. High glucose shows an increasing but not significant signal. Additional treatment with MGO shows a decrease (0.703 ± 0.185; *p* = 0.034) compared to the 5.5 mM glucose control.

### 2.7. Sialic Acid Content Correlates with Glucose Level and Glycation Leads to a Slightly Reduction in Sialic Acid

To investigate whether the observed changes in adhesion and invasion were related to glycosylation, we analyzed the sialic acid content.

**Figure 9:**
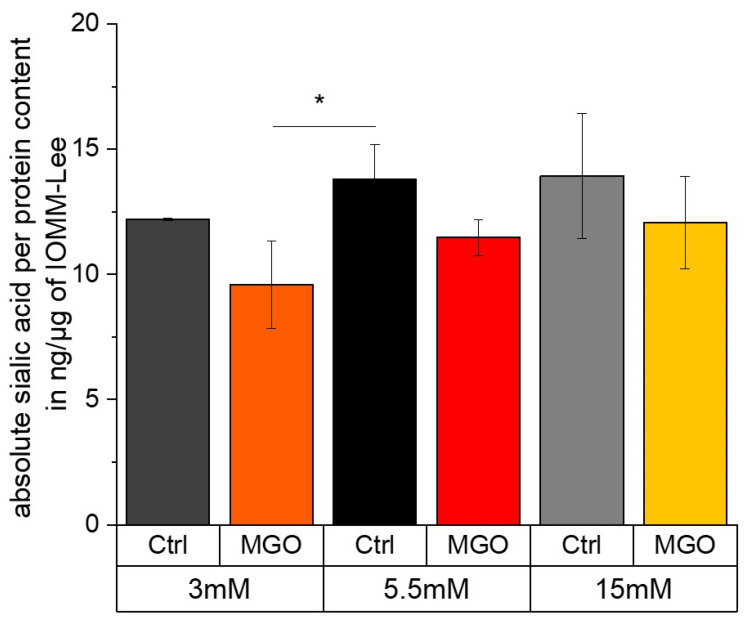
Absolute sialic acid per protein content in ng/µg in different glucose with and without MGO-treated IOMM-Lee cells. Sialic acid per protein content in ng/µg in different glucose levels with and without MGO for 24 h-treated IOMM-Lee cells was measured using HPLC (sialic acid in ng) and BCA (protein conc. in µg/µL). Statistical analysis was performed using *t*-test and error bars represent SD (n = 4; *p* < 0.05 = *).

Figure 9 shows the sialic acid content per protein in ng/µg in normal glucose compared to low and high glucose with and without MGO treatment. In low glucose (12.208 ± 0.044 ng/µg; *p* = 0.14), the sialic acid content is slightly but not significantly decreased compared to normal glucose (13.814 ± 1.388 ng/µg). Normal glucose compared to high glucose without additional MGO treatment (13.937 ± 2.489 ng/µg; *p* = 0.94) shows no significant difference. In general, the sialic acid content decreases with additional MGO treatment. In low glucose with MGO (9.593 ± 1.758 ng/µg; *p* = 0.019), we observed a significantly lower sialic acid content. This lower level is almost significant in normal glucose with MGO (11.478 ± 0.725 ng/µg; *p* = 0.054). Also, in high glucose with MGO (12.078 ± 1.85 ng/µg; *p* = 0.245), we observed a lower but not significant sialic acid content per protein.

## 3. Discussion

Malignant tumors are known to use glucose as their primary energy source [6,7,8]. For this reason, it has been considered to influence the behavior of malignant tumors by means of a glucose-reduced diet. Some studies have shown that fasting can inhibit the progression of tumor cells from various tissues [52,53]. For instance, Ho et al. have shown that a ketogenic diet inhibits tumor growth and prevents the initiation of cancer [54]. Fasting also lowers an abundance of extrinsic and intrinsic growth factors, especially the insulin-like growth factor 1 (IGF-I), which acts as the major growth effector of growth hormone [55]. In mice, a short-term starvation (24–72 h) decreased IGF-I production by 70% which resulted in an 11-fold increase in its counterpart IGF-binding protein 1 (IGFBP-1) [56,57]. The influence of glycation, particularly MGO, has also been investigated in some studies. Depending on the entity studied and the MGO concentration, treatment led to the accumulation of reactive oxygen species (ROS) and DNA damage or apoptosis or, conversely, increased proliferation and invasiveness [58,59,60,61,62]. In the study presented here, we were able to show that low glucose levels of 3 mM led to a significant reduction in cell viability compared to normal (5.5 mM) glucose. With additional treatment with 0.1 mM and 0.3 mM MGO, we observed similar results.

Further, we were able to show that glucose and especially MGO have an influence on the migratory potential of the malignant meningioma cells used. The resistance of the ECIS measurement, which is measured at low frequency, gives an indication of cell–cell contacts. The impedance measurement provides information on adhesion, spreading and confluence and the capacity measurement provides information on spreading and confluence—both performed at high frequency [63]. In our hands, cell–cell contacts are negatively affected by high glucose or normal glucose with additional glycation. This is in line with our own previous work, where we showed that glycation can affect cell–cell contacts in benign and malignant meningioma cells—for example, in benign meningioma cells, glycation led to an increased expression of E-cadherin and a decreased expression of N-cadherin. [49]. Further studies have also shown that glycation can enhance the migratory and invasive potential of tumor cells, which are critical for metastasis and prognosis [64,65,66]. In addition, we observed that normal and high glucose with MGO have negative effects on adhesion, spreading and confluence. This could be a sign of anti-adhesive or anti-proliferative and/or apoptotic behavior [59]. In breast cancer cells, glycation has been shown to lead to increased metastasis through activation of Receptor of AGE (RAGE)/Toll-like receptor 4 (TLR4) signaling, and anti-AGE drugs have been shown to reverse the effects on migratory behavior [66,67]. In lung adenocarcinoma, high glucose promotes tumor cell proliferation and migration via the RAGE-NOXs pathway [68]. Swami and colleagues have shown that glycation, which upregulates RAGE, leads to a decreased expression of FAK signaling pathways in addition to a decreased expression of integrin β1, for example. These observations also led to reduced migratory potential [69].

Subsequently, surface and effector proteins were analyzed for their contribution to migration. At low glucose, with and without additional glycating agent, we observed a decreasing expression of integrin β1. Zhang and colleagues have shown that a high expression of integrin β1 in liver cancer influences metastatic behavior [70]. On the other hand, a study by Mizejewski and colleagues showed that a low expression of integrin ß1 has a cancer-promoting effect [71]. The heterodimer of integrin β1 and α5 analyzed by Miroshnikova et al. found that there is a correlation between the upregulation of α5 β1 integrin and cancer aggression via phosphoinositide 3-kinase (PI3K)-dependent tumor cell invasion [72]. We analyzed integrin α5 but did not observe expression via immunoblot. At high glucose, we observed an upregulation of integrin β1 compared to normal glucose levels. Focal adhesion is impaired by high glucose with MGO, resulting in a decreased expression of FAK and p-FAK. The expression of p-FAK is also downregulated by low glucose and MGO treatment. Human schwannoma cells have been shown to release IGFBP1, which activates Src/FAK signaling in an integrin β1-dependent manner, mediating increased proliferation and cell–matrix adhesion [73]. Poulikakos and colleagues have shown that the re-expression of merlin/NF2 inhibits invasive potential in mesothelioma cells, which was associated with the attenuated phosphorylation of FAK. In addition, it was demonstrated that merlin downregulation resulted in increased invasion [74] and merlin deficiency has an effect on integrin β1-dependent mediated cell–matrix interaction and leads to downstream FAK signaling [75]. In our study, we did not detect any significant changes in merlin/NF2 expression via immunoblot that could affect FAK or integrin β1 expression.

Interestingly, in terms of invasive potential, we saw a decreased but not significant change in low glucose with and without MGO and a slightly increased but not significant change in high glucose. This is consistent with our observations of integrin β1 expression in the immunoblot and sialic acid content in the different glucose levels with and without additional MGO treatment. The high glucose treatment of colorectal tumor shows an increase in proliferation and metastasis via the PI3K/AKT/mTOR pathway [76]. In a previous study, we have shown that glycation in IOMM-Lee cells leads to reduced invasive behavior [49]. Other studies have shown that glycation can lead to increased invasion in different cancer types, which we observed in benign meningioma cells in a previous study [49,77,78]. It has been shown in previous studies that MGO has an effect on the expression of sialyltransferases in various tumor cells and thus may also increase the level of sialic acids [48,79].

More and more evidence suggests that the abnormal glycosylation of glycoproteins, such as truncated and branched glycan structures and sialylated termination increase, are associated with developing malign transformations [43,80,81,82,83,84]. Many studies have shown that abnormal sialylation has an impact on the outcome for the patient, because it directly affects migration, invasive potential, drug resistance and immune escape mechanisms [85,86,87,88]. Wu and colleagues show that sialylation through ST3GAL1 promotes cell migration, invasion and TGF-β1-induced EMT, which results in paclitaxel resistance in ovarian cancer [88]. Additionally, an α2,6 sialylation of β1 integrins promotes integrin-mediated cell migration, adhesion and cell proliferation [89]. A study of O-glycan structures in breast cancer has shown that generally in malignant transformation, tumor cells produce simpler and fewer types of glycan structures [83]. The simplest aberrant O-glycan (Tn antigen) is also a pan-cancer epitope which could suggest the aggressiveness of the disease [90]. The presence of the Tn antigen may inhibit the further elongation of neighboring glycan structures [91]. The sialylation of Tn antigen results in STn and is associated with an increase in metastasis, which could mediate through the reduced interaction of the malignant cell with the tissue resident galacectins [92,93]. Sialylated surface proteins could have an impact in immune escape mechanism due to masking and may hinder physical interactions, e.g., NK-cells [94] or trigger inhibitory signaling through Siglec receptors [95].

Several authors have been able to illustrate that the quantity of sialic acids and the degree of glycosylation are a decisive factor in the aggressiveness of tumor diseases [96,97,98]. In our experiments, the sialic acid content was significantly lower at low glucose levels and glycation. On the other hand, a slight but not significant increase in sialic acid content was observed at high glucose levels. The sialic acid content in our data shown here is slightly lower than in another study using primary tumor cells [99]. Polysialic acid is known to interfere with cell–cell and cell–substrate interactions, playing a role in processes such as adhesion, migration and invasion [89,100,101,102].

A limitation of this work is the use of an immortalized cell line. In the future, an analysis of primary cultures may provide a better understanding of the behavior of these tumors.

## 4. Materials and Methods

### 4.1. Cell Culture

The human malignant meningioma cell line IOMM-Lee (ATCC^®^ CRL-3370™) was obtained from the American Type Culture Collection (ATCC, Manassas, VA, USA). The cell line was cultured in Dulbecco’s modified Eagle’s medium (DMEM) supplemented with 100 µg/mL streptomycin, 100 U/mL penicillin, 4 mM glutamine and 10% fetal bovine serum (FBS, Sigma-Aldrich, St. Louis, MO, USA) at 37 °C in a 5% CO_2_ incubator. The cell line was lysed with 0.1% trypsin-EDTA (ethylenediaminetetraacetic acid) solution for 2 min every 2–3 d [49].

### 4.2. Cell Viability Assay

The cell viability of glycated IOMM-Lee cells was measured using a MTT assay (Sigma Aldrich). Both cell lines were seeded into 96-well plates at a density of 7.8 × 10^4^/cm^2^ cells per well in DMEM containing 1% FBS. After 2 h of attachment, cells were treated with different concentrations of MGO (Sigma Aldrich, 40% aqueous solution; diluted in 1 × PBS; 0.1 mM, 0.3 mM). The controls (Ctrl) were cells without MGO treatment. Cells were cultured for 24 h. MTT was diluted to a final concentration of 0.5 mg/mL in normal growth medium and the cells were incubated for 2 h with 10 μL MTT solution per well. After the removal of the MTT-containing medium, the remaining formazan crystals were dissolved in 150 μL dimethyl sulfoxide (DMSO). Absorbance values were measured (Plate-Reader, Clariostar, BMG Labtech GmbH, Ortenberg, Germany) at a wavelength of 570 nm (background 630 nm). The untreated control cells were set to 1 for cell viability. Changes in the cell viability of the treated cells were calculated relative to the untreated control [49].

### 4.3. Examination of Migration with Electric Cell Substrate Impedance Sensing (ECIS)

The migration of meningioma cells was examined using ECIS arrays (8W1E PET ECIS CulturewareTM, Applied Biophysics Inc., Troy, NY, USA). The arrays were coated with L-cysteine (10 mM) for 10 min. The arrays were then washed twice with deionized water and coated with fibronectin 5 µg/mL for 2 h at 37 °C. Cells were then seeded at a density of 1 × 10^5^/cm^2^. After reaching a capacity of <0.3, the cells were treated with different glucose concentrations of 3 mM, 5.5 mM, 15 mM glucose with and without 0.3 mM MGO. Wounding was initiated after 12 h of culture in treatment media and analyzed for another 12 h after wounding. 

### 4.4. Immunoblot

Cells were seeded in 10 cm dishes at a density of 1 × 10^6^/cm^2^ in DMEM containing 1% FBS. After 2 h of attachment, cells were treated with different concentrations of MGO (0.3 mM). Controls (Ctrl) were cells without MGO treatment. Cell lines were cultured for 24 h. Cells were directly lysed in hot SDS sample buffer (2.5% sodium dodecyl sulfate, 0.06 M TRIS pH 6.8, 10% glycerol, 0.01% brome phenol blue, 10 mM dithiothreitol in TBS-T (TRIS buffered saline 0.1% tween)) to isolate the total protein. Proteins were separated using sodium dodecyl sulfate-polyacrylamide gel electrophoresis (SDS-PAGE, 10%) and transferred to a nitrocellulose membrane using Western blot techniques. Detection of focal adhesion kinase (FAK) and phospho-FAK (p-FAK) expression was performed using polyclonal anti-p-FAK antibody (0.1 µg/mL, Cell Signaling Technology, Boston, MA, USA), polyclonal anti-FAK antibody (0.1 µg/mL, Abcam, Waltham, MA, USA) and a secondary peroxidase-linked antibody (ImmunoResearch Inc., Eagan, MN, USA). To detect integrin β1, a monoclonal primary antibody (integrin beta1 (D2E5) rabbit mAb, cell signaling Technology, Boston, MA, USA) was used. Merlin/NF2 was detected with a monoclonal anti-NF2/merlin antibody (EPR2573(2), Abcam, Waltham, MA, USA). Images were captured using the Chemidoc MP imaging system (Bio-Rad Laboratories, Hercules, CA, USA). Ponceau S staining (0.1% Ponceau S, 3% trichloroacetic acid and 3% sulfosalicylic acid) of the total loaded protein was used as the loading control. The band intensities of the proteins of interest were converted to numerical values using Image lab software (Bio-Rad Laboratories, https://www.bio-rad.com/, accessed on 15 September 2024) and normalized to the corresponding Ponceau S staining to quantify the results [49]. 

### 4.5. Examination of Invasion with Real Time Cell Analysis (RTCA)

Invasion was analyzed in 96X CIM plates (ACEA Biosciences). The CIM plates consist of an upper and a lower chamber. The bottom surface of the upper chamber consists of a microporous membrane through which cells can migrate. On the underside of this membrane, a gold electrode detects the presence of adherent cells. To examine invasion, 800 µg/mL of basement membrane matrix, lactose dehydrogenase elevating virus (LDEV)-free Matrigel^®^ (Corning, Minneapolis, MN, USA) was added to the upper chamber. After incubation for 4 h at 37 °C, 160 µL DMEM with 20% FBS was added to the lower chamber and 50 µL DMEM with 1% FBS was added to the upper chamber. The CIM plates were incubated at 37 °C for 1 h. The background signal was then measured. The cells were trypsinized and detached. The reaction was stopped by adding media containing 1% FBS and the cells were resuspended. Cells were added to the upper chamber at a density of 1.1 × 10^5^/ cm^2^. Invasion was measured as changes in impedance with the RT-CES^®^ system and monitored every 15 min for a period of 48 h. The measurement was performed with the RTCA DP Analyzer (ACEA Biosciences) and displayed with the RTCA program 2.0 (ACEA Biosciences) [49].

### 4.6. Acidic Hydrolysis

Samples were freeze dried. Then, 1 mL of 2 M propionic acid was added, resuspended, vortexed and incubated for 4 h at 80 °C with shaking. The samples were then cooled on ice for 10 min, followed by centrifugation for 20 min, 13,200 rpm at 4 °C. The supernatant was lyophilized overnight. The samples were resuspended in 0.5 mL aqua dest. The protein concentration was then measured twice for each sample [103]. 

### 4.7. DMB Labeling of Sialic Acids

Sample and reference solutions were labeled (2.5 h, 50 °C, shaking) with DMB labeling reagent (1,2-diamino-4,5-methylenedioxybenzene.2HCl (DMB), 1.6 mg/mL, DMB Sialic Acid Labelling Kit, QA Bio, Palm Desert, CA, USA). Samples were diluted 2:1 with water and assayed in duplicate [103].

### 4.8. HPLC

To measure the sialic acid concentration, 20 µL of the labeled sample and reference were injected into the instrument. A LiChroCART^®^250-4 LiChrospher^®^100 RP-18e (5 µm) column (Merck KGaA, Darmstadt, Germany) was used. A reference panel (1.25 nmol, AdvanceBio Sialic Acid reference panel, Agilent, Santa Clara, CA, USA) was used as a reference for each measurement. Solvent A (acetonitrile–methanol–water 9:7:84) and solvent B (acetonitrile) were used at a flow rate of 0.5 mL/min. Concentrations were determined using a diluted standard of the appropriate sialic acid (50 ng/mL to 10 µg/mL, Sigma Aldrich, St. Louis, MO, USA) [103].

### 4.9. Statistical Analysis

All analyses and visualizations were performed using OriginPro 2019 software (OriginLab Corporation, Northampton, MA, USA). A paired Student’s *t*-test was performed against the control group, both cell lines or a theoretical value of 1 (due to data normalization). The figures show the average mean with standard deviation (SD) and the levels of significance are shown within the figures.

## 5. Conclusions

In this study, we have shown that glucose with or without additional glycation has different effects on malignant meningioma cells. Invasive potential was reduced at low glucose levels, which correlated with a reduction in cell viability and a significant decrease in sialic acid levels. High glucose levels with additional glycation also reduced invasive potential, but did not affect sialic acid levels. We also observed an increased invasive potential at high glucose levels, although it was not significant. Barrier function was disrupted and adhesion was reduced at high glucose levels, which is associated with increased migratory potential. Consistent with this, FAK expression was significantly reduced at high glucose levels.

Our data indicate that an intentional low-glycemic diet may improve the outcome of patients with malignant meningioma by positively influencing tumor growth and potential.

## Data Availability

The dataset is available from the corresponding author upon reasonable request.
